# Toward explainable deep learning in healthcare through transition matrix and user-friendly features

**DOI:** 10.3389/frai.2024.1482141

**Published:** 2024-11-25

**Authors:** Oleksander Barmak, Iurii Krak, Sergiy Yakovlev, Eduard Manziuk, Pavlo Radiuk, Vladislav Kuznetsov

**Affiliations:** ^1^Department of Computer Science, Khmelnytskyi National University, Khmelnytskyi, Ukraine; ^2^Department of Theoretical Cybernetics, Taras Shevchenko National University of Kyiv, Kyiv, Ukraine; ^3^Laboratory of Communicative Information Technologies, V.M. Glushkov Institute of Cybernetics, Kyiv, Ukraine; ^4^Department of Mathematical Modeling and Artificial Intelligence, National Aerospace University “Kharkiv Aviation Institute”, Kharkiv, Ukraine; ^5^Institute of Computer Science and Artificial Intelligence, V.N. Karazin Kharkiv National University, Kharkiv, Ukraine

**Keywords:** healthcare, artificial intelligence, deep learning, medical signal processing, medical image analysis, model interpretability

## Abstract

Modern artificial intelligence (AI) solutions often face challenges due to the “black box” nature of deep learning (DL) models, which limits their transparency and trustworthiness in critical medical applications. In this study, we propose and evaluate a scalable approach based on a transition matrix to enhance the interpretability of DL models in medical signal and image processing by translating complex model decisions into user-friendly and justifiable features for healthcare professionals. The criteria for choosing interpretable features were clearly defined, incorporating clinical guidelines and expert rules to align model outputs with established medical standards. The proposed approach was tested on two medical datasets: electrocardiography (ECG) for arrhythmia detection and magnetic resonance imaging (MRI) for heart disease classification. The performance of the DL models was compared with expert annotations using Cohen’s Kappa coefficient to assess agreement, achieving coefficients of 0.89 for the ECG dataset and 0.80 for the MRI dataset. These results demonstrate strong agreement, underscoring the reliability of the approach in providing accurate, understandable, and justifiable explanations of DL model decisions. The scalability of the approach suggests its potential applicability across various medical domains, enhancing the generalizability and utility of DL models in healthcare while addressing practical challenges and ethical considerations.

## 1 Introduction

The rapid development of AI has made it important to explain the decisions made by AI systems, a concept known as explainable artificial intelligence (XAI) (Confalonieri et al., 2020). Within AI, machine learning (ML) encompasses various algorithms and models, including traditional ML methods and DL techniques. DL, a subset of ML, utilizes neural networks with multiple layers to model complex patterns in data. However, DL models often suffer from the “black box” problem, where their internal decision-making processes are not transparent, limiting their trustworthiness in critical applications like healthcare (Hassija et al., 2024).

It is also worth noting that XAI implements the “right to explanation” (Vredenburgh, 2022), that is, the right to have a clear explanation of the result of the algorithm’s work. This right applies to each of us when the algorithm’s decision directly affects a person. Such rights are already being developed, although the general «right to explanation» is still under discussion. In the information society, the “right to explanation” is becoming an extremely important concept, as digital technologies, AI, and ML will continue to be actively applied to solving various problems of human activity (Venkatesan et al., 2023; Longo et al., 2024).

Our study enhances the role of AI in resource distribution and strategic decision-making by making DL model decisions more interpretable for healthcare providers. This interpretability is crucial for effective decision-making and resource management in health crises like the COVID-19 pandemic (Zaoui et al., 2023). Moreover, the paper addresses the ethical, legal, and societal dimensions of AI by emphasizing transparency and trustworthiness in AI applications. The proposed methods ensure that AI decisions are accurate, understandable, and justifiable by establishing clear criteria and metrics. We define “understandable” as the degree to which healthcare professionals can comprehend the model’s decision-making process through interpretable features that are directly related to clinical knowledge. “Justifiable” refers to the model’s ability to provide explanations that are supported by clinical guidelines and empirical evidence. These criteria are quantitatively assessed using statistical metrics such as Cohen’s Kappa coefficient to measure agreement between model explanations and expert annotations, and by evaluating the consistency of the model’s decisions with established medical standards.

In this study, we aim to address the issue of explaining decisions made by AI. Previously, in Radiuk et al. (2024), we proposed an approach to explain the results of a DL model by mapping its decisions to those of a traditional ML model using a transition matrix. This approach requires both a DL model and a corresponding ML model trained on the same data. However, in practice, we often have a DL model without an equivalent ML model. In this case, we cannot apply the specified approach to explaining the decisions made by the DL model.

Therefore, the objective of this study is to apply our approach to the case of medical data processing, where there is no ML model corresponding to the DL model. Instead, we consider a set of features that are understandable to healthcare experts. Using these features, we propose interpreting the decisions obtained by the DL model. To fulfill the study’s goal, it is essential to develop a new scalable visual analytics approach. The scalability of our approach lies in its ability to be applied across different types of medical data and tasks without the need for retraining the DL model or developing new ML models for each case. By utilizing a transition matrix to bridge the DL model’s decision-making with expert-defined features, the method can be adapted to various medical signal and image processing applications. This adaptability allows for efficient extension to new datasets and clinical problems, thereby enhancing its practical utility in diverse healthcare settings. Finally, the main contribution of this work is the scalable approach to explain the results obtained by DL models, based on features understandable to healthcare experts for medical signal and image processing tasks.

The structure of the article is as follows. The following section presents an analysis of the current state of the problem under study. Section 3 describes the proposed scalable approach to presenting decisions made by DL models using features understandable to the physician, given the solution of medical signal and image processing problems. Section 4 presents the results of computational experiments and their interpretation. Finally, Section 5 concludes the results obtained and suggests further directions of this research.

## 2 State of the arts

In general, it is believed that XAI adheres to three principles: transparency, interpretation, and explanation (Phillips et al., 2021). We can talk about the inherent transparency of XAI if the developer can describe and explain how the model forms and updates parameters from statistical training data and how it makes predictions on new data (Pääkkönen and Ylikoski, 2021). By interpretation of XAI, we mean understanding how the AI model forms its output data and explaining its decisions to people (Räuker et al., 2023). Explanation in XAI is an important concept but without a clear definition. It is believed that AI explanation in a broad sense is a set of features that influenced the decision (i.e., classification or prediction) for a specific case (Notovich et al., 2023). If AI-based approaches meet these requirements, then they are said to provide the basis for justifying decisions, tracking and verifying them, and improving and researching new facts (Kim et al., 2023).

Explainable artificial intelligence issues are especially critical in areas such as medicine, defense, finance, and law, where it is important to understand AI decisions and trust them (Manziuk et al., 2021; Mora-Cantallops et al., 2024). Today there are many approaches that provide decent results in various tasks of such sensitive areas of human activity (Wang and Chung, 2021). In general, DL methods provide better results compared to traditional ML methods for solving problems with heterogeneous data (Krak et al., 2023). In particular, convolutional neural network (CNN) models (Radiuk et al., 2021) are state-of-the-art for computer vision tasks (Smith et al., 2021), and transformer models are state-of-the-art for natural language processing tasks (Khurana et al., 2023). However, as already mentioned, decisions made by DL methods are not always transparent and understandable.

The field of XAI is experiencing significant advancements, particularly in the development of methods to enhance the transparency of AI models in the healthcare domain. Researchers are actively exploring various approaches, including the construction of feature models and the use of manually crafted features to provide clearer explanations of AI decisions. As an example, Bassiouny et al. (2021) present an innovative approach to diagnosing neonatal lung diseases by training an object detection model, faster-RCNN, to identify seven key lung ultrasound features rather than making direct diagnostic predictions. This methodology enhances the interpretability of the results and keeps clinicians in control by providing annotated images to support their diagnostic decisions. The study demonstrates that the model surpasses single-stage detectors like RetinaNet, achieving high mean average precision, thus balancing performance with trustworthiness in medical practice.

In their review, Salahuddin et al. (2022) explore various interpretability methods for deep neural networks in medical image analysis, emphasizing that these methods aim to enhance transparency and trust in AI systems. They highlight that while these interpretability techniques provide valuable insights, they are often approximations and may not fully capture the true decision-making processes of the models, necessitating cautious application in clinical settings. In addition, Chan et al. (2022) developed and compared three ML models to predict long-term mortality in critically ill ventilated patients, finding that boosting algorithms and logistic regression achieved similar performance.

Similarly, Lu et al. (2023) propose a comprehensive workflow that includes a step where medical professionals label differential diagnosis features according to medical guidelines, effectively blacklisting irrelevant features extracted from electronic health records. This approach aims to “reduce workloads of clinicians in human-in-loop data mining” by focusing on feature oversight rather than full prediction, thus enhancing the trustworthiness and efficiency of the AI model.

In Moreno-Sánchez (2023), a heart failure survival prediction model is enhanced by integrating explainable AI techniques, aiming to balance predictive performance and interpretability. This approach provides transparency by explaining feature contributions to predictions, making the model’s decision-making process clearer for clinicians. Consequently, it fosters greater trust and practical adoption in clinical settings.

Pintelas et al. (2023) introduce a novel framework for 3D image recognition that utilizes interpretable features such as lines, vertices, and contours to enhance explainability. This approach is particularly promising for medical imaging, achieving performance comparable to state-of-the-art black-box models while maintaining transparency. However, the development of interpretable methodologies for 3D image segmentation remains an emerging area of research, with most existing techniques originally designed for 2D image classification tasks.

Based on the analysis of existing literature, we identified a lack of clear methodologies for constructing feature models that enhance the interpretability of DL models in medical applications. The primary goal of this study is to enhance the decision-making processes of DL models in processing medical signals and images by introducing a novel scalable approach that translates complex model outputs into interpretable features understandable to healthcare professionals.

The main scientific contributions of this work are:

- We introduced a new scalable visual analytics approach that utilizes a transition matrix to bridge the DL model’s decision-making with interpretable features defined by experts.
- Our approach systematically incorporates clinical guidelines and expert rules into the feature selection and model development process.
- We applied and validated our approach on two distinct medical datasets–ECG signals for arrhythmia detection and MRI scans for heart disease classification–achieving strong agreement with expert annotations (Cohen’s Kappa coefficients of 0.89 and 0.80, respectively).

## 3 Materials and methods

### 3.1 Basic approach

**Figure 1:**
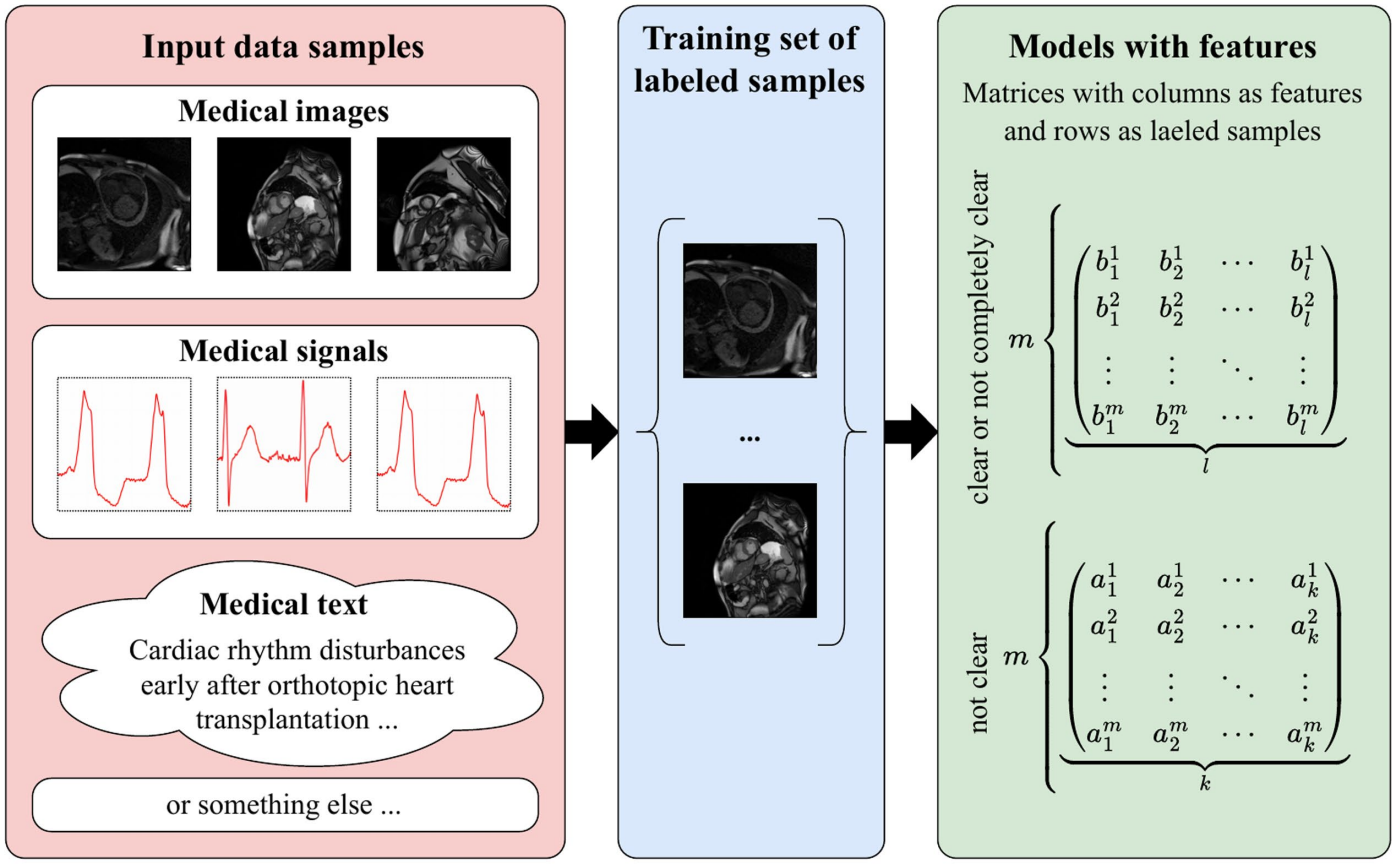
The basic idea of our approach: The process of converting various medical data types–such as images, signals, and text–into a labeled training dataset, which is subsequently transformed into feature matrices where rows represent labeled samples and columns correspond to extracted features, organized by clarity for model development.

In Radiuk et al. (2024), we addressed the problem of explaining decisions made by DL models by establishing a relationship between the features learned by a DL model and those used in a traditional ML model. This idea is illustrated in Figure 1.

The process described above involves the formation of ML models, which have all the necessary features of understandable AI: transparency, interpretability, and explainability. Otherwise, these features (areas of attention) are formed according to certain algorithms (DL models) and, as a result, are not entirely clear, or not at all clear to the end user.

It is worth noting that there are also intermediate cases when the signs are “in the middle” between the indicated cases, such as:

- Decomposition of understandable areas of attention into “incomprehensible” signs, both with the possibility of reverse transformation and without such possibility.
- In addition to features understandable to the public of experts, there may be separate features (or combinations of previously obtained ones) that are understandable to more experienced experts or are based on intuition; here, for each specific case, the community decides whether these cases are transparent or not.

As a result of the above, let us have one training sample and two models with features. One model is built by the DL model, and the other model has features formed by an expert.

Next, we formalize the problem under consideration. We represent the features of the DL model in the form of matrix *A* of dimension *m* × *k* and the features of the other model in the form of matrix *B* of dimension *m* × *l* as follows:


(1) $$\begin{array}{l} m\{\underset{k}{\underset{︸}{\left(\begin{array}{cccc} a_{1}^{1} & a_{2}^{1} & \cdots & a_{k}^{1}\\ a_{1}^{2} & a_{2}^{2} & \cdots & a_{k}^{2}\\ \cdots & \cdots & \cdots & \cdots \\ a_{1}^{m} & a_{2}^{m} & \cdots & a_{k}^{m} \end{array}\right)}}=A \end{array}$$

(2) $$\begin{array}{l} m\{\underset{l}{\underset{︸}{\left(\begin{array}{cccc} b_{1}^{1} & b_{2}^{1} & \cdots & b_{l}^{1}\\ b_{1}^{2} & b_{2}^{2} & \cdots & b_{l}^{2}\\ \cdots & \cdots & \cdots & \cdots \\ b_{1}^{m} & b_{2}^{m} & \cdots & b_{l}^{m} \end{array}\right)}}=B \end{array}$$

where *m* is the number of vectors obtained from the training sample during DL model training, *k* represents the number of features, and *l* stands for the number of features.

We emphasize once again that the features formalized in formulas (1, 2) are obtained from the same training sample. We also note that in general *k* can be equal to, less than, or greater than *l*.

In practical problems that are modeled in this way, that is, in the presence of two mappings for the same objects for different sets of features, it is often necessary to express feature vectors of different dimensions through each other. In other words, consider the problem where for different matrices *A* and *B* it is necessary to find such a matrix *T* that the following equality holds:


(3) B=TA

where *T* is the transition matrix between matrices *A* and *B*.

Note that in linear algebra, formula (3) is a usual change of basis of a vector space, and if the condition *m* = *k* = *l* is met, finding matrix *T* is trivial, that is:


(4) $$T=BA^{-1}$$

For the case under consideration, *m* ≠ *k* ≠ *l*, the inverse matrix does not exist, and therefore it is proposed to apply a generalization of the inverse matrix—the pseudo-inverse matrix (Cvetković Ilić and Wei, 2017). We propose to find such a matrix *T* of dimension *k* × *l*, which provides the transition between matrices *A* and *B*:


(5) AT≈B

Note that the approximation in formula (5) is established with respect to the Euclidean norm in the feature space of the matrices. It is proposed to find matrix *T* as follows:


(6) $$T\approx A^{+}B$$

In practice, it is proposed to define $A^{+}$ using SVD decomposition (Kalman, 2002), even though other methods are described in Krak et al. (2020):


(7) $$A^{+}=V\Sigma^{+}U^{T}$$

where $A=V\Sigma \;U^{T}$ is the singular value decomposition of matrix *A*, the matrix $\Sigma^{+}$ is formed by transposing the matrix Σ and replacing all its non-zero values of diagonal elements with inverse ones:


$$\begin{array}{c} \Sigma^{+}=\left[D^{+}\vdots 0\right],\;m\geq k;\\ \;\Sigma^{+}=\left[\begin{array}{c} D^{+}\\ \cdots \\ 0 \end{array}\right],\;m<k;\\ D^{+}=\mathrm{diag}\left(\sigma_{1}^{+},\sigma_{2}^{+},\sigma_{3}^{+},\ldots ,\sigma_{t}^{+}\right),\;\sigma_{i}^{+}=\{\begin{array}{c} \frac{1}{\sigma_{i}^{+}},\sigma_{i}>0;\\ 0,\sigma_{i}=0. \end{array} \end{array}$$

Therefore, for an arbitrary row vector of features $a_{j}^{*}$ , $i=\bar{1,k}$ , obtained by the model defined by matrix *A*, the corresponding row vector of features $b_{i}^{*}$ , $j=\bar{1,l}$ , by the model defined by matrix *B*, is determined using the obtained transition matrix *T* as follows:


(8) $$b_{i}^{*}=a_{j}^{*}T,\;i=\bar{1,k},\;j=\bar{1,l}$$

The approach described above by formulas (1–8) somehow correlates with approximation, that is, the description by one function, even given in tabular form, of a given form of another function, perhaps also in tabular form.

There are several approaches to data approximation. One of them consists in approximating a complex function with a simpler function, which is used for all tabular values, but it is not necessary that it passes through all points. This approach is also called curve fitting, which is sought to be carried out so that its deviation from the tabular data is minimal. The authors propose to use the transition matrix *T* according to formula (4) between two feature models, presented in the form of matrices, for the same set of input data as such a function.

**Figure 2:**
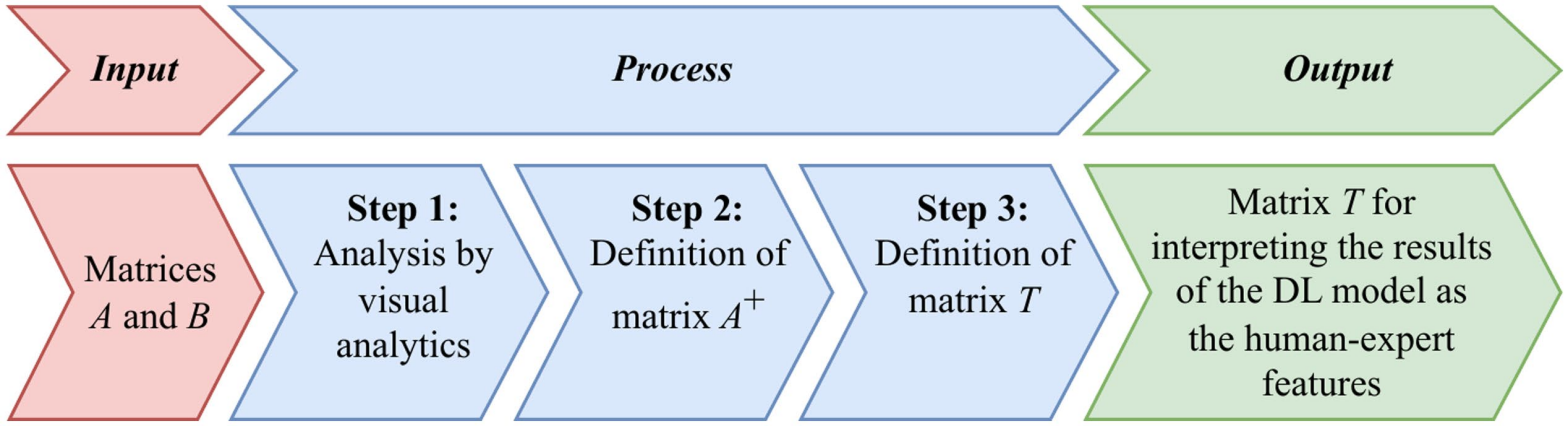
Diagram of the basic approach to derive the transition matrix *T* from input matrices *A* and *B*, involving three main steps: visual analytics of the matrices, defining matrix *A*^+^, and establishing matrix *T*, which serves to interpret DL model outputs in terms of human-expert features.

Figure 2 briefly shows the main steps of the basic approach, first proposed in Radiuk et al. (2024) to obtaining the transition matrix *T*.

First, we extract two matrices, *A* from the DL model and *B* from the ML model, both representing the same data samples.

*Step 1:* Use a visual analytics tool, either Principal Component Analysis (PCA) by Pearson (1901), or *t*-distributed Stochastic Neighbor Embedding (*t*-SNE) by Hinton and Roweis (2002), to map these high-dimensional feature vectors onto a two-dimensional plane.
A dimensionality reduction technique is aimed at preserving the local structure of the data and reveal clusters, allowing us to visually compare the feature vectors from the DL model and the interpretable feature set. By ensuring their relative positions match across models, we facilitate the accurate computation of the transition matrix *T*.

*Step 2:* Compute the pseudo-inverse matrix $A^{+}$ .

*Step 3:* Calculate the transition matrix *T*.

Finally, use matrix *T* to translate DL results into features understandable by the ML model.

### 3.2 The proposed scalable approach

To overcome the absence of a corresponding ML model, we propose a scalable approach that constructs matrix B using expert-defined interpretable features. This approach allows us to apply the transition matrix method to enhance the interpretability of DL models.

The proposed scalable approach is aimed at simplifying complex, hard-to-understand features from a DL model into a more user-friendly form, making the results easier to interpret. The extracted feature vector, which is the penultimate layer in a DL model, is transformed using a transition matrix *T* by formula (6) to produce results that are understandable to the end user.

Suppose there is an expert in the subject area of the problem under consideration (i.e., the end-user) who compiles an exhaustive list of features by which they determine the belonging of an object to a particular class. Further, for each feature from the list of features, the expert indicates the numerical intervals into which the value of the feature should fall for the classes under consideration. Finally, for each instance (object) from the training dataset, the value of each feature is calculated.

The values of features can be determined in several ways, namely:

- Empirically, using the expert’s knowledge of the subject area of the problem under consideration.
- Using formulas or statistical indicators that are understandable to the end user.
- By visual representation (in various ways) of a fragment of a signal or image, in comparison with similar fragments from labeled training data.
- Utilizing visual analytics.
- Using ML models specially built for this case.
- Using DL models specially built for this case.

The selection of interpretable features is guided by the following criteria:

- Clinical relevance: Features must be directly related to clinical indicators that healthcare professionals use for diagnosis and treatment decisions.
- Measurability: Features should be quantifiable using available tools or methods, ensuring consistent measurement across different samples.
- Distinctiveness: Selected features should provide unique information about the data, minimizing redundancy and multicollinearity.
- Expert Consensus: Features should be agreed upon by a panel of experts to reflect standard clinical understanding and practices.

**Figure 3:**
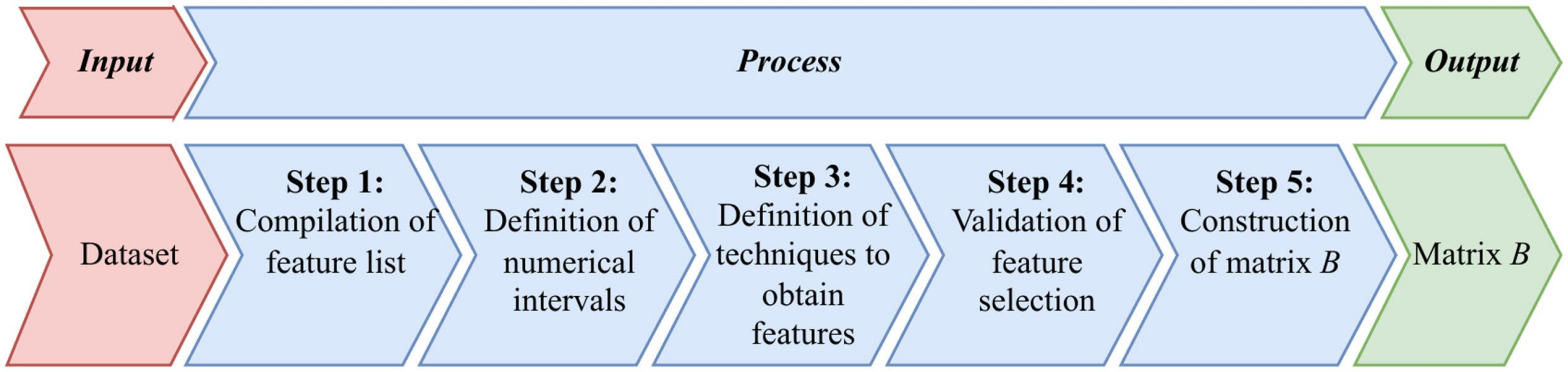
Diagram that outlines the proposed sequential approach to constructing matrix *B* from an initial dataset, through steps including compiling a feature list, defining numerical intervals, selecting techniques to extract features, validating feature selection, and ultimately constructing matrix *B* based on the defined transition matrix from the deep learning model to the feature model.

Figure 3 shows the main steps of the method for constructing matrix *B*, according to the proposed scalable approach.

Below we provide the following steps to build matrix *B*.

Input information: An expert panel in the subject area of the problem under consideration and the dataset on which the DL model was trained.

*Step 1:* Compilation of feature list. An expert panel comprising cardiologists and medical imaging specialists compiles a comprehensive list of interpretable features based on clinical guidelines, literature, and diagnostic practices. The goal is to cover all relevant aspects that could influence the classification while ensuring the features meet the criteria above.

*Step 2:* Definition of numerical intervals. For each feature, the experts define numerical intervals that correspond to different classes or pathological conditions. These intervals are based on clinical thresholds and empirical data, providing a clear delineation between classes.

*Step 3:* Definition of techniques to obtain features. We establish standardized methods for quantifying each feature:

- Direct measurement: Using signal processing techniques or image analysis tools to extract quantitative values from the data.
- Computational algorithms: Applying validated algorithms (e.g., peak detection algorithms for ECG) to automate feature extraction.
- Statistical analysis: Employing statistical methods to calculate features such as mean values, variances, and ratios that are clinically significant.

*Step 4:* Validation of feature selection. The selected features undergo validation to ensure reliability and consistency:

- Pilot testing: Features are tested on a subset of the data to assess their discriminative power and measurement consistency.
- Inter-rater reliability: Multiple experts independently measure the features on the same samples to calculate agreement levels, using statistical metrics.
- Refinement: Based on validation results, features may be refined, or additional features may be included to enhance interpretability and accuracy.

*Step 5:* Construction of matrix *B*. Using the validated methods, we extract feature values for each sample in the training set, forming matrix *B*. This matrix represents the data in terms of interpretable features aligned with expert understanding.

Output information: Matrix *B*.

It should be also noted that the scalability within the proposed scalable approach refers to the following characteristics:

- Adaptability to various medical domains: The method can be efficiently extended to different types of medical data and DL models without substantial changes to the core methodology.
- Ease of integration with expert-defined features: By utilizing a transition matrix and mapping DL outputs to interpretable features, the approach can be applied across different clinical problems with minimal adjustments.

### 3.3 Evaluation criterion

In this work, Cohen’s Kappa coefficient (*κ*) is used to evaluate the quality of the proposed approach. The *κ* coefficient is a reliable statistical indicator for evaluating inter-expert reliability for qualitative (categorical) elements. It quantifies the level of agreement between two experts beyond chance.

The formula for Cohen’s Kappa coefficient *κ* is as follows:


(9) $$\kappa =\frac{P_{o}-P_{e}}{1-P_{e}}$$

where $P_{o}$ is the level of observed (empirical) agreement between two experts, and $P_{e}$ is the level of expected (calculated) agreement between the same experts.

For the problem of binary classification of medical signals and/or images with a confusion matrix consisting of true positives (TP), false positives (FP), true negatives (TN), and false negatives (FN), the elements of formula (9) have the following form:


(10) $$P_{o}=\frac{TP+TN}{TP+FP+TN+FN}$$

(11) $$P_{e}=\left(\frac{\left(TP+FP\right)\times \left(TP+FN\right)+\left(TN+FN\right)\times \left(TN+FP\right)}{{\left(TP+FP+TN+FN\right)}^{2}}\right)$$

In formula (10), $P_{o}$ is the proportion of cases in which the DL model and the human expert come to a consistent decision. Instead, in formula (11), $P_{e}$ is calculated based on the marginal sum values of the decisions of the two experts.

The value of the *κ* coefficient according to formula (9) is in the range [−1;1], where 1 denotes perfect agreement, 0 stands for no agreement, and negative values reflect less agreement than expected by chance.

## 4 Results and discussion

Experimental evaluation of the proposed scalable approach was performed by solving two problems with DL models:

- ECG analysis: Utilized a deep CNN based on the architecture proposed by Kovalchuk et al. (2024).
- MRI analysis: Employed another deep CNN with a U-Net architecture for image segmentation and classification as described in Slobodzian et al. (2023).

Next, we present the results and discussion of the application of the proposed approach to explaining the decisions made by DL models.

### 4.1 Detection of pathologies of heart activity based on ECG

The proposed approach was validated by the constructed ${DL}_{ECG}$ model for the problem of detecting pathologies of heart activity (arrhythmias) based on ECG in Kovalchuk et al. (2024). Below we describe the training dataset, the ${DL}_{ECG}$ model, and the set of features that explained the decisions and results of the proposed approach (the value of *κ*).

#### 4.1.1 Training dataset and DL model

The problem of detecting pathologies of heart activity (arrhythmias) based on ECG was solved using the reference dataset MIT-BIH Arrhythmia Database (MIT-BIH) (Moody and Mark, 2005). The training of the ${DL}_{ECG}$ model was performed on 80% of the data from MIT-BIH. Given the annotations of the MIT-BIH set, the following classes/pathologies were selected for the classification problem:

- Normal beat.
- Premature ventricular contraction.
- Paced beat.
- Right bundle branch block beat.
- Left bundle branch block beat.
- Atrial premature beat.
- Fusion of ventricular and normal beat.
- Fusion of paced and normal beat.
- Others.

**Figure 4:**
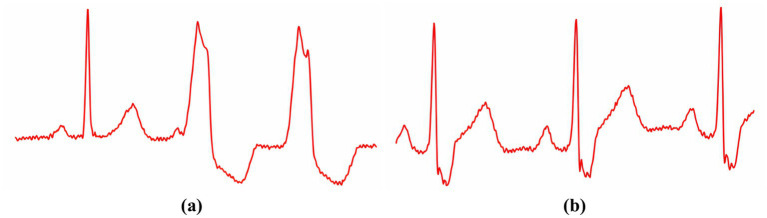
This figure presents an ECG signal fragment displaying a triad of cardiac cycles, where panel (A) illustrates a heterogeneous appearance of the R peak, while panel (B) shows a homogeneous expression of the R peak across cycles.

Input information for training and testing the ${DL}_{ECG}$ model is presented as a triad of cardiac cycles—in the center is the main cardiac cycle, to which the previous and next cardiac cycles were added (Figure 4).

In this work, the ${DL}_{ECG}$ model was created based on the modified architecture from Kovalchuk et al. (2024). The classification accuracy for the training set was 99.95%, for the test set—99.13%.

The penultimate layer of the ${DL}_{ECG}$ model contained 8,192 neurons, and the number of samples in the training sample was 52,180. Accordingly, the size of the $A_{ECG}$ matrix was $m_{ECG}$ = 52,180—the number of objects from the training subsample of the MIT-BH dataset, $k_{ECG}$ = 8,192—the number of features formed by the ${DL}_{ECG}$ model.

#### 4.1.2 Features on ECG for explanation

For the experiment, we focused on detecting PVCs or Ventricular Extrasystole as defined by established clinical guidelines, such as the American Heart Association’s recommendations on ECG interpretation. Cardiologists integrated these guidelines to identify key ECG features for PVC detection, applying expert rules to enhance model explanations and performance:

1. Absence of the P wave:

- Integration of clinical guidelines: According to clinical standards, the absence of a P wave preceding a QRS complex suggests ectopic ventricular activity, characteristic of PVCs.
- Expert rules applied: If the P wave is absent or not temporally associated with the QRS complex, it indicates a PVC.
- Method of measurement: Used the NeuroKit2 toolkit (Makowski et al., 2021) to detect P wave presence, ensuring compliance with guidelines for accurate P wave identification.

1. Expanded and deformed QRS complex:

- Integration of clinical guidelines: Clinical guidelines state that PVCs present with widened (≥120 ms) and abnormally shaped QRS complexes due to aberrant conduction pathways.
- Expert rules applied: A QRS duration exceeding 120 ms with atypical morphology is indicative of a PVC.
- Method of measurement: Employed a shallow neural network trained on data annotated per these guidelines to detect QRS abnormalities.

1. Full compensatory pause:

- Integration of clinical guidelines: A full compensatory pause following a PVC is a diagnostic criterion, where the sum of the pre-and post-PVC RR intervals equals twice the normal RR interval.
- Expert rules applied: Applied the rule that $RR_{\mathrm{prev}}+RR_{\mathrm{next}}\approx 2\times {RR}_{n}$ , within a clinically acceptable tolerance.
- Method of measurement: Calculated RR intervals using NeuroKit2, adhering to guidelines for RR interval measurement. In the following subsection, we provide a detailed description of the measurement process.

By integrating clinical guidelines and expert rules into feature selection and measurement, we enhanced the model’s explanations and performance, aligning the DL model’s outputs with clinical practice.

#### 4.1.3 Statistical analysis for ECG classification

Given the significant amount of training and the significant time of experts regarding filling in the values of features, non-empirical methods of determining the value of features were used in this work.

For the “Absent P peak” feature, PCA was used. The application of PCA and the reduction of data dimension to 3 made it possible to make sure that the signal fragment with the presence and absence of P peaks is separate. Given this, the presence/absence of the P peak was determined using the Neurokit2.

**Figure 5:**
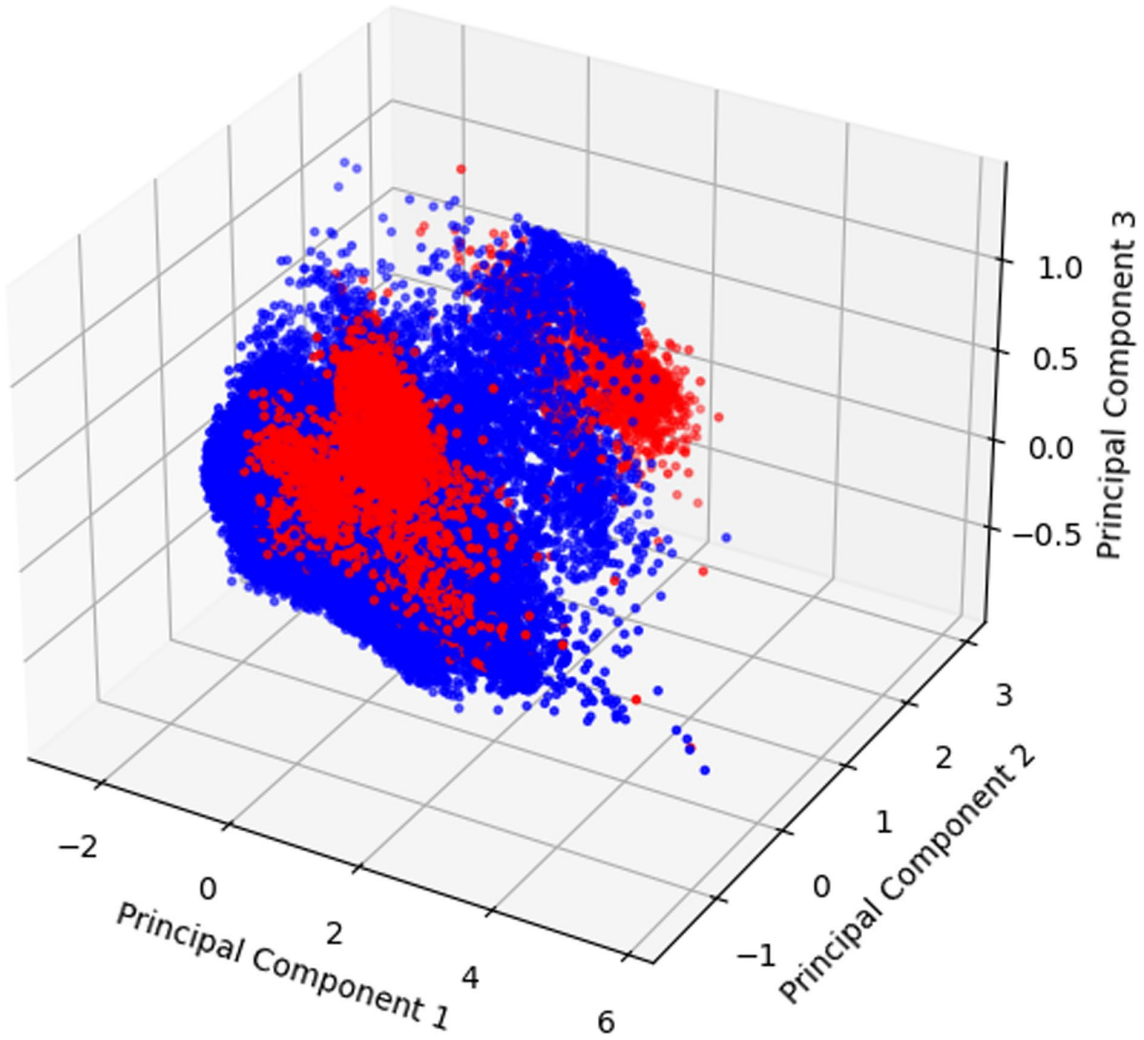
The results of applying PCA to classify data based on the “Absent P peak” feature, showing data points distributed across three principal components for visualization of feature separation.

Visualization of dimensionality reduction by PCA for the “Absent P peak” feature is shown in Figure 5.

For the “Expanded and deformed QRS complex” feature, due to the complexity of its detection by other methods, it is proposed to use a specially trained neural network.

The “Full compensatory pause” feature. A compensatory pause is the time elapsed after an extrasystole until the occurrence of a normal contraction. Therefore, in the case when the extrasystole is located between other extrasystoles, this calculation is not performed and is calculated only for the last case of extrasystole in the sequence.

The presence or absence of this feature was checked as follows:

- Using the Neurokit2 package, the average RR interval between normal cardiac cycles ( ${RR}_{n}$ ) was determined.
- The interval between the R peak with extrasystole and the R peak of the previous cycle ( ${RR}_{\mathrm{prev}}$ ) was determined.
- The interval between the R peak with extrasystole and the R peak of the next normal cycle ( ${RR}_{\mathrm{next}}$ t) was determined.

A full compensatory pause was determined under the following conditions:


$$\begin{array}{c} {RR}_{\mathrm{prev}}+{RR}_{\mathrm{next}}=2\times {RR}_{n},\\ \left(2\times {RR}_{n}-\left({RR}_{\mathrm{prev}}+{RR}_{\mathrm{next}}\;\right)\right)<\mathrm{tolerance} \end{array}$$

According to the above rules, the values of features were determined for each sample from the training set, and, in this way, matrix *B* was obtained. Further, according to formula (6), the transition matrix *T* was determined.

Coefficient $\kappa_{1}$ was calculated to evaluate the agreement between the class annotations in the test set and the class predictions made by the ${DL}_{ECG}$ model. Coefficient $\kappa_{2}$ was calculated to determine the agreement between the class annotations obtained by the ${DL}_{ECG}$ model and those obtained by the approximated feature values.

The resulting $\kappa_{1}$ was 0.98. To assess the precision of this estimate, a 95% confidence interval (CI) was computed, resulting in a CI of 0.96–1.00. Additionally, the associated *p* value was calculated to be <0.001, indicating that the observed agreement is highly unlikely to be due to chance. The high $\kappa_{1}$ value, combined with the narrow confidence interval, signifies an almost perfect agreement between the expert annotations and the ${DL}_{ECG}$ model’s predictions. This strong agreement is further supported by the *p* value, which confirms the statistical significance of the result.

The resulting $\kappa_{2}$ was 0.89, with a 95% CI of 0.85–0.93, and a *p* value of <0.001. This $\kappa_{2}$ value indicates a strong agreement between the model’s predictions and the approximated feature-based annotations, although it is slightly lower than $\kappa_{1}$ . The slightly broader confidence interval reflects a bit more variability in the agreement, which could be attributed to the approximation process. Nonetheless, the *p* value still indicates that this agreement is highly significant, and the $\kappa_{2}$ value demonstrates that the expert’s features can reliably replicate the decisions made by the ${DL}_{ECG}$ model.

The comparison between $\kappa_{1}$ and $\kappa_{2}$ , along with their respective confidence intervals and *p*-values, provide a comprehensive understanding of the model’s performance. The high $\kappa_{1}$ value, coupled with a narrow confidence interval and a significant p-value, confirms the ${DL}_{ECG}$ model’s capability to accurately classify ECG in alignment with expert annotations. The slightly lower, yet still strong, $\kappa_{2}$ value suggests that while the approximated feature values can effectively mirror the model’s decisions, there is a slight decrease in agreement, which may warrant further investigation into the approximation methods, or the features used.

### 4.2 Detection of pathologies of heart activity based on MRI

The proposed scalable approach was also validated by the ${DL}_{MRI}$ model for the problem of detecting pathologies of heart activity based on MRIs (Slobodzian et al., 2023).

Next, we briefly describe the training dataset of MRIs, the ${DL}_{MRI}$ model, the set of features that explained the decisions, and the results of the proposed approach (the value of *κ*).

#### 4.2.1 Training dataset and DL model

For the problem of detecting pathologies of heart activity based on MRIs, a modified dataset of the Automatic Cardiac Diagnosis Challenge (ACDC) (Bernard et al., 2018) was used. Samples of the ACDC set of 100 and 50 patients were used for training and testing the network, respectively. Given the annotations to the ACDC set, the following classes/pathologies were selected for classification:

- Normal condition.
- Dilated cardiomyopathy (DCM).
- Hypertrophic cardiomyopathy (HCM).
- Myocarditis (MINF).
- Arrhythmogenic right ventricular cardiomyopathy (ARV).

**Figure 6:**
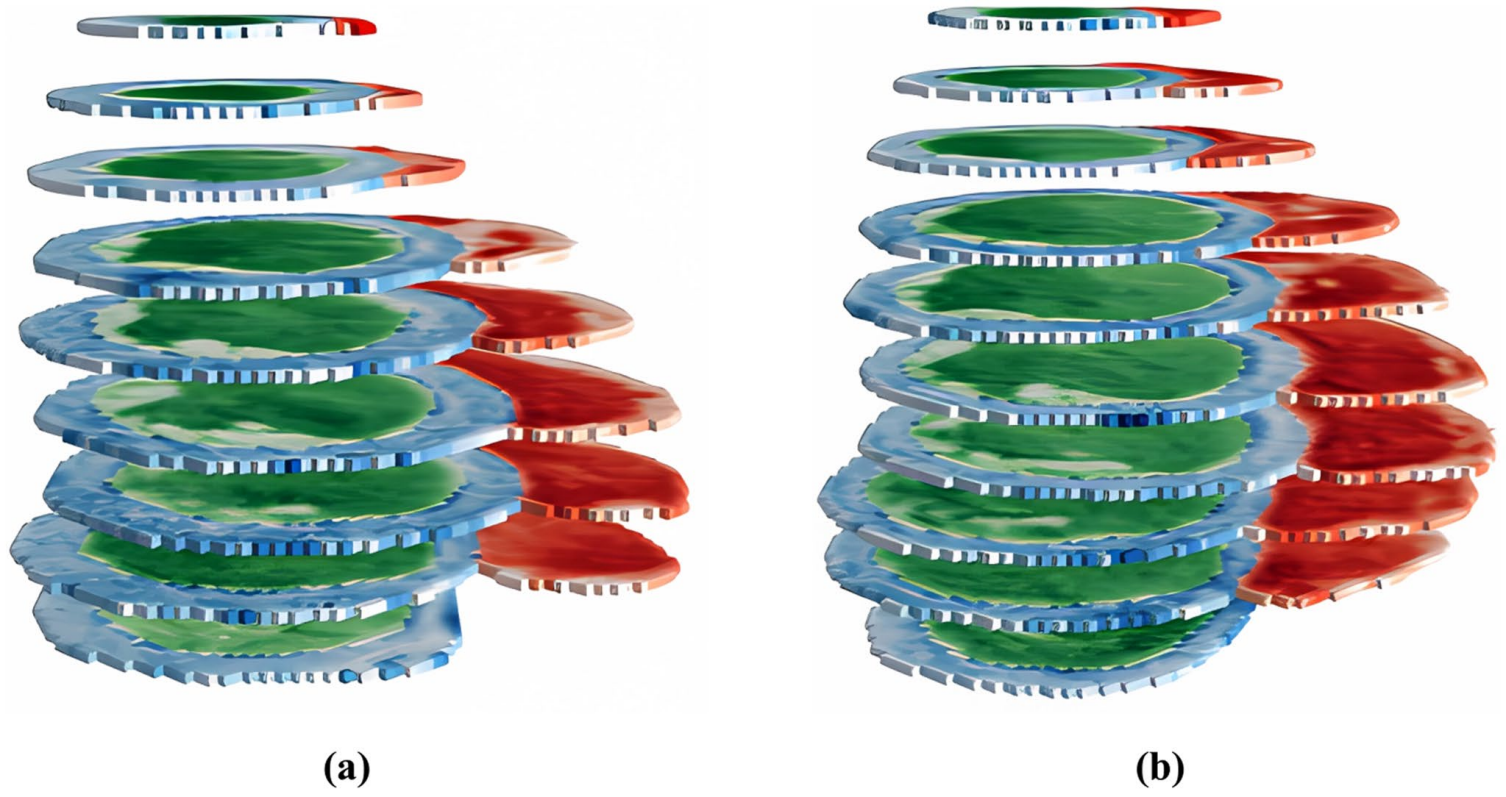
A 3D visualization of segmented MRI data prepared for classification, with images from the ES phase in panel (A) and the ED phase in panel (B), while preserving MRI signal intensity values across layers for enhanced phase differentiation.

An example of presenting input data to the DL model according to the ACDC dataset is illustrated in Figure 6.

The ${DL}_{MRI}$ model was created based on the modified architecture from Slobodzian et al. (2023). The classification accuracy for the test set of MRIs was over 96.5%. The size of the $A_{MRI}$ matrix was $m_{MRI}$ = 100 – the number of objects from the training subsample of the ACDC dataset, $k_{MRI}$ = 1,024 – the number of features formed by the ${DL}_{MRI}$ model.

#### 4.2.2 Features in MRI for explanation

For the classification task, 20 features were considered. At the same time, according to the features of identifying pathologies identified by the doctor, the following set of geometric features was formed for further classification:

- The ratio of the volume of the left ventricle to the volume of the right ventricle at the end of systole.
- The volume of the left ventricle at the end of the systole.
- The ratio of the volume of the left ventricle to the volume of the right ventricle at end-diastole.
- The volume of the left ventricle at end-diastole.
- The volume of the right ventricle at end-systole.
- The volume of the right ventricle at end-diastole.
- Ejection fraction of the left ventricle.
- Ejection fraction of the right ventricle.
- The ratio of myocardial volume to left ventricular volume at the end of systole.
- Myocardial mass at the end of diastole.
- Myocardial volume at the end of systole.
- The ratio of myocardial mass to left ventricular volume at the end of diastole.
- Maximum average myocardial wall thickness at end-diastole.
- Maximum average myocardial wall thickness at end systole.
- Mean standard deviation of myocardial wall thickness at end-systole.
- Mean standard deviation of myocardial wall thickness at end-diastole.
- Standard deviation of the standard deviation of myocardial wall thickness at end-diastole.
- Standard deviation of the standard deviation of myocardial wall thickness at end-systole.
- Standard deviation of mean myocardial wall thickness at end-diastole.
- Standard deviation of mean myocardial wall thickness at end-systole.

For the interpretation task, we utilized 20 features selected based on clinical guidelines for diagnosing DCM, such as those provided by the American College of Cardiology and the European Society of Cardiology. Cardiologists integrated these guidelines and applied expert rules to identify key geometric features, enhancing model explanations and performance:

1. Left ventricular end-diastolic volume (LVEDV):

- Integration of Clinical Guidelines: Elevated LVEDV is a primary diagnostic criterion for DCM according to clinical guidelines.
- Expert Rules Applied: An LVEDV exceeding clinically established thresholds, adjusted for body surface area, indicates DCM.
- Method of Measurement: Calculated LVEDV from segmented MRI images using volumetric analysis, following standardized protocols.

1. Left ventricular ejection fraction (LVEF):

- Integration of Clinical Guidelines: Reduced LVEF (<45%) is a key indicator of systolic dysfunction in DCM patients.
- Expert Rules Applied: An LVEF below clinical thresholds signifies impaired cardiac function consistent with DCM.
- Method of Measurement: Derived from LVEDV and end-systolic volume (LVESV) using the formula:

$$\mathrm{LVEF}=\frac{\mathrm{LVEDV}-\mathrm{LVESV}}{\mathrm{LVEDV}}\times 100\%\text{.}$$

1. Myocardial mass at end-diastole:

- Integration of Clinical Guidelines: Changes in myocardial mass are significant in DCM diagnosis, as per clinical standards.
- Expert Rules Applied: Increased myocardial mass beyond normal ranges for a given body size indicates pathological remodeling associated with DCM.
- Method of Measurement: Estimated myocardial mass using standardized techniques, ensuring compliance with clinical measurement guidelines.

By incorporating clinical guidelines and expert rules into the feature selection and quantification process, we ensured that the features are clinically meaningful, thereby enhancing the interpretability and performance of the ${DL}_{MRI}$ model.

#### 4.2.3 Statistical analysis for MRI classification

For further experiments, the DCM class was chosen. Since the volume of the training set was small, it did not take much time for experts to fill in the values of features. Cardiologists as experts determined the values of features for each sample from the training set and, in this way, matrix *B* was formed. Further, according to formula (6), the transition matrix *T* was determined.

For each object from the test set, the values of features were approximated according to formula (8). Subsequently, two key values of *κ* were also calculated to assess the agreement between the predictions ${DL}_{MRI}$ of and the expert annotations. These *κ* values provide a quantitative measure of the reliability and consistency of the ${DL}_{MRI}$ model’s performance, and they were computed using formula (9).

Coefficient $\kappa_{1}$ was calculated to evaluate the agreement between the class annotations in the test set and the classifications made by the ${DL}_{MRI}$ model.

Coefficient $\kappa_{2}$ was calculated to determine the agreement between the class annotations obtained by the ${DL}_{MRI}$ model and those obtained through the approximated feature values.

The resulting $\kappa_{1}$ value was 0.87, indicating a very good level of agreement. To further substantiate this finding, a 95% confidence interval was computed, yielding a range of 0.83–0.91. Additionally, the associated *p*-value was determined to be <0.001, confirming the statistical significance of this agreement. The relatively narrow confidence interval suggests that the $\kappa_{1}$ value is a stable estimate, and the low *p*-value strongly supports that the observed agreement is unlikely due to random chance. This high $\kappa_{1}$ value aligns well with the model’s overall performance, highlighting the ${DL}_{MRI}$ model’s robust capability in accurately classifying MRI data per expert labels.

The resulting $\kappa_{2}$ value was 0.80, with a 95% confidence interval of 0.76–0.84, and a *p* value of <0.001. This $\kappa_{2}$ value indicates a significant match, though slightly lower than $\kappa_{1}$ , reflecting a strong agreement but with slightly more variability. The broader confidence interval compared to $\kappa_{1}$ suggests some degree of uncertainty, possibly arising from the approximation process or the inherent variability in the feature values. Nonetheless, the significant *p* value still confirms that this agreement is meaningful and not due to random variation.

The comparison between $\kappa_{1}$ and $\kappa_{2}$ , along with their respective confidence intervals and *p*-values, provides a detailed insight into the ${DL}_{MRI}$ model’s performance and the reliability of the feature approximation approach. The $\kappa_{2}$ value of 0.87, with a narrow confidence interval and a highly significant p-value, underscores the strong alignment between the model’s predictions and expert annotations. Meanwhile, the slightly lower $\kappa_{2}$ value of 0.80 suggests that while the approximation method is effective, there is a minor decrease in agreement that could be attributed to the complexity of the MRI data or the approximation method itself.

Overall, the inclusion of these detailed statistical indicators, i.e., *κ* values, confidence intervals, and *p* values, adds robustness to the analysis, strengthening the reliability and validity of both ${DL}_{MRI}$ and ${DL}_{ECG}$ model’s performance and the transparency of the proposed scalable approach.

### 4.3 Discussion and limitations of the proposed scalable approach

While the proposed scalable visual analytics approach has demonstrated high reliability and enhanced interpretability by showing strong agreement between the ${DL}_{ECG}$ and ${DL}_{MRI}$ models and expert annotations, several practical challenges and limitations must be acknowledged when considering real-world healthcare applications.

Firstly, the heterogeneity inherent in real-world healthcare settings presents a significant limitation. Diverse patient populations, varying data quality, and differing clinical protocols across institutions can affect the generalizability and robustness of the model. Implementing the approach across multiple institutions may be challenging due to inconsistencies in data formats, acquisition techniques, and labeling standards. This diversity can lead to models that are tailored to specific datasets and may not perform effectively on unseen data from different sources or populations. Variations in patient demographics and disease prevalence can also impact on the model’s ability to generalize, potentially limiting its clinical utility. Addressing this drawback requires extensive validation and potential customization for each setting, which can be resource-intensive.

Secondly, integrating the proposed method into existing clinical workflows poses practical challenges. The approach requires careful planning to ensure it does not disrupt standard practices or burden healthcare professionals. Clinicians may need additional training to interpret the model outputs effectively, and the time required to compute interpretable features and generate explanations must be minimized to be practical in fast-paced clinical environments. This limitation could hinder the adoption of the approach, as any increase in workload or decrease in efficiency is a significant drawback in clinical settings where time and resources are limited. Future research should focus on streamlining the computation processes and developing user-friendly interfaces to facilitate seamless integration.

Data privacy and security concerns present another critical limitation. Utilizing patient data for model training and feature extraction raises significant privacy issues, and ensuring compliance with regulations such as HIPAA (Modifications to the HIPAA Privacy, Security, Enforcement, and Breach Notification Rules, 2013) or GDPR (European Parliament and Council of the European Union, 2016) is essential. There is a risk that the transparency provided by the approach may inadvertently reveal sensitive information or biases present in the data. This limitation is a significant drawback because it can lead to ethical and legal consequences if patient confidentiality is compromised. Implementing secure data handling protocols, anonymization techniques, and establishing ethical guidelines for the use of AI-generated explanations are necessary steps to mitigate these concerns.

The reliance on expert-defined features introduces potential biases and inconsistencies, which is another notable limitation. Creating a comprehensive and universally accepted list of interpretable features is challenging, as experts may have differing opinions on which features are most relevant. This subjectivity can lead to models that only partially capture the diversity of clinical presentations and may overlook subtle but significant patterns present in the data. Additionally, features may exhibit high multicollinearity, where multiple features are correlated with each other, reducing the clarity and effectiveness of the model’s explanations. This limitation can result in oversimplified diagnoses and potentially unreliable model explanations, which is a drawback for clinical decision-making. To resolve this, future research should incorporate data-driven feature selection methods alongside expert input, such as PCA, t-SNE or mutual information techniques, to identify relevant features that enhance the model’s robustness.

Consistency in determining numerical feature values is also a limitation. Experts may struggle to quantify features invariably, especially those requiring subjective assessment or intricate measurement protocols. This inconsistency can introduce variability into the training data, leading to potentially unreliable model explanations and affecting the model’s performance on new data. This is a drawback because it reduces trust in the system’s outputs and can hinder clinical adoption. Developing clear guidelines and automated measurement tools can reduce variability in feature quantification. Leveraging objective, reproducible measurement techniques minimizes reliance on subjective assessments, enhancing consistency across diverse users and settings.

The inherent complexity of DL models poses challenges for interpretability, despite using the transition matrix approach. High-dimensional feature spaces and nonlinear relationships make it challenging to interpret how input data influences the model’s predictions fully. This complexity can hinder the ability to provide clear and actionable explanations to healthcare professionals, which is a significant drawback since interpretability is crucial for trust and acceptance in clinical practice. Utilizing model-agnostic interpretability tools, such as SHapley Additive exPlanations (SHAP) values or Local Interpretable Model-agnostic Explanations (LIME), can provide insights into model predictions even in complex models. Simplifying model architectures where possible and focusing on key features can make interpretations more accessible to clinicians.

Finally, the scalability of our approach suggests its potential applicability to a wide range of medical domains beyond ECG and MRI analysis. By mapping complex DL model outputs to interpretable features defined by experts, the method can be adapted to other types of medical data, such as histopathology images, genomic data, or medical text analysis. For instance, in histopathology, features like cell morphology, tissue patterns, and staining intensities could be used to explain DL models classifying cancer subtypes. In genomics, gene expression levels, mutation frequencies, or pathway activations might serve a similar purpose. Moreover, the approach could help interpret DL models processing clinical notes in medical text analysis by linking model outputs to medically relevant terms and concepts.

Addressing these limitations requires a multidisciplinary effort involving clinicians, data scientists, ethicists, and policymakers. We can enhance the model’s robustness and interpretability by incorporating data-driven feature selection methods alongside expert input, implementing regularization techniques, and utilizing model-agnostic interpretability tools. Developing clear guidelines and automated tools for consistent feature quantification will improve reliability. Establishing ethical frameworks and ensuring compliance with data privacy regulations will mitigate legal and ethical concerns. Enhancing the scalability and generalization of the approach through flexible frameworks and adaptation techniques is essential for its practical implementation. Future research should focus on these areas to overcome the identified drawbacks and facilitate broader implementation of the approach in real-world healthcare applications.

## 5 Conclusion

In this study, we introduced a scalable approach designed to make DL model decisions more explainable by mapping them to interpretable features defined by healthcare experts. The criteria for selecting these features were clearly established, integrating clinical guidelines and expert rules to ensure that the features are clinically relevant, measurable, distinctive, and agreed upon by professionals. The approach was rigorously tested on two distinct medical datasets: ECG signals for detecting arrhythmias and MRI scans for classifying heart diseases. The DL models achieved Cohen’s Kappa coefficients of 0.89 for the ECG and 0.80 for the MRI datasets, demonstrating strong agreement with expert annotations. These results underscore the reliability of the proposed method in providing accurate, understandable, and justifiable explanations of DL model decisions.

Addressing potential limitations, our approach acknowledges the challenges of feature selection biases, generalization to unseen data, and interpretability in complex models. By incorporating data-driven feature selection methods alongside expert input–such as PCA, t-SNE or mutual information techniques–we can reduce biases and enhance the model’s robustness. Implementing regularization techniques, cross-validation, and testing on external datasets can improve generalizability. Utilizing model-agnostic interpretability tools like SHAP values or LIME can provide insights even in complex models, making interpretations more accessible to clinicians.

Overall, our scalable approach enhances the interpretability of DL models in medical applications by providing accurate, understandable, and justifiable explanations according to established medical standards. This positions the method as a valuable tool for integrating AI into diverse areas of healthcare, potentially improving diagnostics, treatment planning, and patient outcomes across various specialties while addressing practical challenges and ethical considerations.

Future work should focus on integrating clinical guidelines and expert rules more systematically into the feature selection and model development process. This integration will enhance model explanations and performance by ensuring that the features and model outputs align with established medical standards. Moreover, improving the feature selection process through standardized and automated methods, along with enhancing the scalability of the approach for adaptation to various medical datasets and clinical environments, will further strengthen the utility and applicability of the method in real-world healthcare settings.

## Data Availability

The original contributions presented in the study are included in the article/supplementary material, further inquiries can be directed to the corresponding author.
